# Structural Validation and Measurement Invariance Testing of the Chinese Version of the eHealth Literacy Scale Among Undergraduates: Cross-Sectional Study

**DOI:** 10.2196/48838

**Published:** 2023-12-13

**Authors:** Chen Long, Lin Zheng, Runhua Liu, Zhongxian Duan

**Affiliations:** 1 Health Services Management Department Guizhou Medical University Guiyang China; 2 Department of Social Welfare Jeonbuk National University Jeonju Republic of Korea; 3 Center of Medicine Economics and Management Research Guizhou Medical University Guiyang China; 4 School of Public Management Guizhou University Guiyang China

**Keywords:** eHealth literacy, eHEALS, factor structure, measurement invariance, undergraduates, health literacy, cross-sectional survey, digital health literacy, measurement

## Abstract

**Background:**

The eHealth Literacy Scale (eHEALS) was introduced in China in 2013 as one of the most important electronic health literacy measurement instruments. After a decade of development in China, it has received widespread attention, although its theoretical underpinnings have been challenged, thus demanding more robust research evidence of factorial validity and multigroup measurement properties.

**Objective:**

This study aimed to evaluate the Chinese version of the eHEALS in terms of its measurement properties.

**Methods:**

A cross-sectional survey was conducted in a university setting in China. Item statistics were checked for response distributions and floor and ceiling effects. Internal consistency reliability was confirmed with Cronbach α, split-half reliability, Cronbach α if an item was deleted, and item-total correlation. A total of 5 representative eHEALS factor structures were examined and contrasted using confirmatory factor analysis. The study used the item-level content validity index (I-CVI) and the average of the I-CVI scores of all items on the scale to assess the content validity of the dominance model. Furthermore, the validated dominance model was subsequently used to evaluate the relevance and representation of elements in the instrument and to assess measurement invariance across genders.

**Results:**

A total of 972 respondents were identified, with a Cronbach α of .92, split-half reliability of 0.88, and item-total score correlation coefficients ranging from 0.715 to 0.781. Cronbach α if an item was deleted showed that all items should be retained. Acceptable content validity was supported by I-CVIs ≥0.80. The confirmatory factor analysis confirmed that the 3-factor model was acceptable. The measurement model met all relevant fit indices: average variance extracted from 0.663 to 0.680, composite reliability from 0.810 to 0.857, chi-square divided by the *df* of 4.768, root mean square error of approximation of 0.062, standardized root mean squared residual of 0.020, comparative fit index (CFI) of 0.987, and Tucker-Lewis index of 0.979. In addition, the scale demonstrated error variance invariance (Δnormed fit index=−0.016, Δincremental fit index=−0.012, ΔTucker-Lewis index=0.005, Δcomparative fit index=−0.012, Δrelative fit index=0.005, and Δroot mean square error of approximation=0.005).

**Conclusions:**

A 3-factor model of the Chinese version of the eHEALS fits best, and our findings provide evidence for the strict measurement invariance of the instrument regarding gender.

## Introduction

### Background

Health-related information is one of the most frequently searched topics on the internet [1,2]. Moreover, health literacy is emerging as a critical factor influencing health outcomes [3-9]. eHealth has emerged as a cost-effective approach that not only plays a key role in expanding the geographic accessibility of secondary prevention programs [10] but also represents a shift to a model of active patient engagement rather than passive acceptance [1].

China’s recently announced 14th Five-Year Plan [11] and long-term goals to 2035 [12] aim to create a “Healthy China” by promoting “Internet+Precision Health Science.” A health science data platform will address individuals’ varying health needs throughout their lifetime. Providing electronic health information that meets the public’s needs and eHealth literacy levels is crucial for informed decision-making [13]. Therefore, developing a tool that comprehensively assesses people’s ability and literacy in comprehending and using web-based information and services is of utmost importance.

Norman and Skinner [14] developed the eHealth Literacy Scale (eHEALS) in 2006, the first assessment instrument to examine this concept. The eHealth literacy concept draws its theoretical foundation from the social cognitive theory and self-efficacy theory by Bandura [15]. These theories emphasize the significance of cognitive processes in learning and behavior changes. As articulated when their Lily model was developed, using social cognitive theory and self-efficacy theory as a conceptual foundation emphasizes that eHealth literacy places more emphasis on promoting competence and confidence than on directly measuring skills with a view to obtaining behavioral change and skill development [14]. Individuals learn and master new behaviors through observation, imitation, and reinforcement. They also develop self-efficacy beliefs about their ability to use these skills effectively, and this belief influences their motivation and ability to engage in certain behaviors. From the perspective of social cognitive theory, behavioral competence refers to the knowledge and skills needed to influence behaviors [16,17].

eHEALS has been translated and adapted for a total of 16 different languages [18] and has shown strong internal consistency and retest reliability across a wide range of age groups, from 12 to 91 years, in over 10 countries [19]. Research on the applicability of eHEALS in China began in 2013 [20]. Over the past decade, this measure has undergone extensive development and is no longer limited to high school students but has been successfully expanded to patients [21], older adults [22], and rural communities [23]. Furthermore, it has been validated as an ideal tool for assessing eHealth among diverse populations. The intracity traffic ban implemented in China during the COVID-19 pandemic posed a significant challenge to the health care system [24]. Consequently, the public has turned to alternative strategies, such as using social media platforms such as WeChat and Dou Yin (ie, TikTok) [25] as well as other digital health technologies [26], to obtain health care information and support and seek web-based medical assistance [27].

With the interest in this area, some specific research focuses have emerged on the psychometric properties of eHEALS. The first pertains to the factor structure of these instruments. For instance, an initial validation study of the eHEALS scale offered evidence that it measures a unidimensional construct [11], commonly referred to as the Lily model [28]. To the best of our knowledge, there are at least 11 factor structures of eHEALS to data [18,29-36]. However, evidence from published studies suggests that the single- and 2-factor structures of eHealth literacy instruments have controversial issues in confirmatory factor analysis (CFA), including poor fit indexes [33,34,37]. The adoption of a multifactorial structure has garnered considerable attention as an effective approach to comprehensively capture the multifaceted nature of eHealth literacy conceptualization. Two-factor structures tend to successfully capture dimensions that emphasize information search and communication skills and use of web-based health resources [29-31], which echoes the central types of literacies analytic and context-specific proposed by Norman and Skinner [28].

However, the two 3-factor structures of eHEALS, namely, awareness, skills, and evaluation, proposed by Sudbury-Riley et al [33], and information awareness, information seeking, and information engagement for eHealth information, developed by Paige et al [35], have received much attention in recent years because they are based on solid research evidence [18], and both the evaluate subdimension [33] and the information engagement subdimension [35] go beyond the individual’s knowledge and perception of behavioral competence and are directly related to self-efficacy. Although some studies conducted in China have confirmed the unidimensionality of eHEALS using exploratory factor analysis [38,39], prior evidence suggests that more complex structural equation models should be used to construct the CFA of this critical measurement instrument, as has been done in other language versions of eHEALS [40-44].

The second major research focus pertains to measurement invariance or equivalence, which is crucial for meaningful group comparisons. Specifically, it is important to establish measurement invariance, as measurement instruments designed for one culture or population may not necessarily measure the same constructs in another culture or population [45]. Examining the significance of measurement invariance when comparing eHEALS across cultures has been a long-standing concern that merits greater attention [32,46-48]. From previous research evidence, measurement invariance studies of eHEALS in other language settings have been particularly interested in participant antecedents, such as gender, age, and education level [42-44]. If the research basis for establishing measurement invariance is neglected, studies and comparisons of differences in access to and use of eHealth resources by different groups may produce ambiguous and erroneous results [33,35,42,45,49]. Although there have been reports of measurement invariance for eHEALS in recent years, to our knowledge, studies conducted in Chinese populations lack exploration of multifactor structures [20,23,39,50], particularly 3-factor structures [38]. Furthermore, there is a lack of evidence for invariance measurements, which are not helpful for the promotion and in-depth study of eHEALS in China.

### Objectives

The purpose of this study was to examine the reliability of the version of the Chinese version of eHEALS (C-eHEALS) and its ability to differentiate extreme scale subjects. A comparison of the 5 models identified superior ones. Content validity and convergent validity were used to assess the appropriateness and relevance of the indicators, respectively. Cross-gender measurement invariance was examined for potential score differences because of structural factors or confounding variables.

## Methods

### Recruitment

A cross-sectional study was conducted at 2 universities in Guizhou Province, China. This study enrolled 1044 undergraduates. This study’s data were collected during late September and early November 2021. The study used a hybrid sampling approach combining convenience and snowball sampling methods to overcome their limitations and create a more diverse participant cohort. After obtaining informed consent, participants were given the choice to complete the paper-and-pencil survey provided by their school counselor teacher or via Wen Juan Xing (a Chinese web questionnaire platform). Of the final 972 valid questionnaires (93.1% response rate), 98.3% (956/972) of participants chose the electronic survey, and 1.6% (16/972) completed the paper-and-pencil survey.

### Ethics Approval

This study was approved by the Human Trials Ethics Committee of Guizhou Medical University (2021-LUNSHENDI-150).

### Instruments

The following sociodemographic variables were measured across the sample: (1) gender (man or woman), (2) age (in years), (3) grade (freshman or sophomore and above), (4) body weight and height (to calculate the BMI), and (5) highest educational attainment of the mother (elementary school and below, junior high school, high school or secondary school, and junior college and above); (6) eHealth literacy was measured by the most widely recognized C-eHEALS [20]. The scale used in this study consists of 8 items, each of which is rated on a 5-point Likert-type scale that ranges from “strongly disagree” to “strongly agree.” The C-eHEALS and sociodemographic question items are presented in Multimedia Appendix 1.

### Statistical Analysis

#### Overview

CFA was performed with Amos (version 24.0; IBM Corp), the rest was performed with SPSS (version 24.0; IBM Corp). The data set is reported in Multimedia Appendix 2. Response distribution and floor and ceiling effects were used to describe the response pattern. Participant characteristics and comparisons of scores across groups were described as percentages and means (SD), respectively. The internal consistency reliability was estimated using Cronbach α and split-half reliability. Item-total correlations were also considered, as well as the α, if item deleted. Structural validity and convergent validity will be used to assess models with 5 different factor structures: the unidimensional structure of Norman and Skinner [28], 2-factor structure from Richtering et al [30] and Soellner et al [29], and the 3-factor structure by Sudbury-Riley et al [33] as well as Paige et al [35]. In addition, 3 analytic invariance tests were performed within the CFA framework to test whether the instrument functions similarly across genders.

#### Item Statistics and Internal Consistency Reliability

The response distribution for each item was analyzed for extreme item deficits at the end of the scale, which could limit the instrument’s responsiveness. The criterion for floor and ceiling effects was that more than 15% of respondents achieved the lowest (strongly disagree) or highest scores (strongly agree), respectively [51].

Cronbach α >.70 is considered good, whereas a split-half reliability >0.70 is acceptable. Item-total correlations >0.70 are acceptable [52].

#### Construct Validity

CFA was used to test the structure of the C-eHEALS. Model fit was determined by the following indicators: chi-square/*df* (*χ*^2^/*df*) ≤5 [53,54], root mean square error of approximation (RMSEA) ≤0.08 (this was considered fair), comparative fit index (CFI), Tucker-Lewis index (TLI) ≥0.95 (indicating a good model fit), and a standardized root mean squared residual (SRMR) ≤0.08 (considered as acceptable) [55].

#### Convergent Validity

Whether each item is closely related to its expected hypothesis structure (convergent validity) was tested by assessing factor loadings, average variance extracted (AVE), and composite reliability (CR). A total of 3 indicators of convergent validity—the standardized factor loadings of each latent variable—should be >0.50 in the first step, the AVE value should be >0.50 in the second step, and the CR should be >0.70 in the third step [56].

#### Content Validity

We used the item-level content validity index (I-CVI) to quantify the content validity of the target model. In addition, the average of the I-CVI scores of all items on the scale (S-CVI/Ave) was used to assess the adequacy of the item representation within the construct’s content domain. The set criteria for I-CVI were ≥0.78 [57], and for S-CVI/Ave, it was ≥0.90 could be considered evidence of good content validity [57,58].

#### Measurement Invariance

The analysis follows the technique suggested by Byrne [59], with subsequent extensions. The first test was for configural invariance (configural model), in which no constraints were applied across the groups. The second test (measurement model) is used to test metric invariance and investigate whether the factor loadings are constrained to be equal across both groups. If they are equal, it can be assumed that the scale intervals are the same in both groups. The third test (structural model) found group equivalence with constrained factor loading parameters and equality constraints on the factor variances and covariances. Finally, the most restrictive test examined measurement error variance invariance, with additional equal error variances across observed variables, to test C-eHEALS’s gender measurement invariance.

Model comparisons were performed using a chi-square test to test measurement invariance at each level and several model fit indices, such as normed fit index (NFI), incremental fit index (IFI), TLI, CFI, relative fit index (RFI), and RMSEA, to evaluate the fit of the final model. These increasingly restrictive models were tested in terms of the fit of the data to the model [60]. Invariance across subgroups is indicated by significant changes in model fit, and because the dependence of ΔCFI and ΔRMSEA on sample size is small, these 2 indices should not exceed 0.02 and 0.015 [61]. The null hypothesis of no difference in model comparisons was accepted if the increase in NFI, IFI, RFI, and TLI values was <0.05 [62].

## Results

### Participant Characteristics

The characteristics of the participants showed that the study had a larger participant base of women (590/972, 60.7%); most were freshmen (705/972, 72.5%) and had a BMI of ≤23.99 kg/m^2^ (853/972, 87.8%). In addition, about half of the participants’ mothers had an education level of elementary school or below (Table 1).

**Table 1:** Summary of participant characteristics (N=972).

			Total, n (%)		Item score, mean (SD)														
					Item 1^a^		Item 2		Item 3		Item 4		Item 5		Item 6		Item 7		Item 8
**Gender**																			
	Man	382 (39.3)		3.58 (0.76)		3.60 (0.79)		3.63 (0.80)		3.62 (0.83)		3.63 (0.82)		3.59 (0.79)		3.54 (0.83)		3.48 (0.79)	
	Woman	590 (60.7)		3.58 (0.79)		3.56 (0.81)		3.64 (0.80)		3.60 (0.79)		3.71 (0.74)		3.60 (0.76)		3.59 (0.77)		3.44 (0.79)	
**Age (y)**																			
	≤19	544 (56)		3.66 (0.76)		3.66 (0.80)		3.72 (0.80)		3.68 (0.81)		3.78 (0.74)		3.68 (0.77)		3.70 (0.78)		3.54 (0.80)	
	20-21	310 (31.9)		3.51 (0.79)		3.48 (0.80)		3.55 (0.78)		3.55 (0.79)		3.59 (0.77)		3.51 (0.77)		3.45 (0.76)		3.38 (0.75)	
	≥22	118 (12.1)		3.40 (0.79)		3.42 (0.77)		3.45 (0.76)		3.42 (0.80)		3.45 (0.81)		3.41 (0.75)		3.28 (0.81)		3.27 (0.79)	
**Grade**																			
	Freshman	705 (72.5)		3.66 (0.75)		3.64 (0.80)		3.70 (0.79)		3.66 (0.80)		3.74 (0.75)		3.66 (0.77)		3.64 (0.80)		3.51 (0.80)	
	Others^b^	267 (27.5)		3.38 (0.81)		3.40 (0.79)		3.45 (0.79)		3.47 (0.80)		3.50 (0.79)		3.42 (0.77)		3.40 (0.76)		3.30 (0.75)	
**BMI (kg/m^2^)**																			
	≤23.99	853 (87.8)		3.56 (0.78)		3.56 (0.80)		3.63 (0.80)		3.61 (0.80)		3.68 (0.76)		3.58 (0.77)		3.55 (0.78)		3.45 (0.78)	
	≥24 to 27.99	119 (12.2)		3.70 (0.79)		3.65 (0.80)		3.67 (0.78)		3.63 (0.83)		3.67 (0.82)		3.67 (0.82)		3.69 (0.85)		3.53 (0.85)	
**Highest education attainment of mother^c^**																			
	Level 1	508 (53.2)		3.56 (0.77)		3.56 (0.80)		3.63 (0.80)		3.62 (0.83)		3.67 (0.78)		3.57 (0.77)		3.54 (0.80)		3.43 (0.80)	
	Level 2	278 (29.1)		3.58 (0.80)		3.62 (0.77)		3.65 (0.80)		3.59 (0.79)		3.65 (0.76)		3.62 (0.79)		3.59 (0.79)		3.50 (0.80)	
	Level 3	93 (9.7)		3.66 (0.80)		3.56 (0.81)		3.65 (0.83)		3.65 (0.83)		3.71 (0.76)		3.65 (0.76)		3.62 (0.77)		3.53 (0.77)	
	Level 4	76 (8)		3.57 (0.74)		3.54 (0.84)		3.63 (0.78)		3.61 (0.75)		3.75 (0.75)		3.67 (0.76)		3.66 (0.78)		3.43 (0.75)	

^a^Item 1 in Chinese eHealth Literacy Scale.

^b^Others: sophomores and above.

^c^Missing data=17 (17 reported that they did not know the highest educational attainment of their mothers). Level 1, elementary school and below; level 2, junior high school; level 3, high school or secondary school; and level 4, junior college and above.

### Score Comparison Across Groups

All groups had mean scores of ≥3.0 or higher, with scores between genders being very close on most items, and men and women having the same mean score on item 1 (Table 1). Participants aged ≤19 years had the highest mean scores across all items (from 3.54 to 3.78), followed by participants aged 20 to 21 years (from 3.38 to 3.59) and then by participants aged ≥22 years (from 3.27 to 3.45). Similarly, freshmen scored higher than sophomores and above on all items. Participants with a BMI between 24 and 27.99 had higher mean scores for all items compared with those with a BMI ≤23.99 kg/m^2^, and only slightly lower mean scores for item 5. The score differences were most pronounced for items 1 and 7, with the smallest differences for item 5. The highest mean scores for all items were found in the group in which the mother’s highest level of education was high school or secondary school.

### Response Distributions

In general, the responses to this tool were not evenly distributed across all levels. For all items, level 1 (strongly disagree) had the lowest percentage of responses, with item 4 and item 7 having the lowest percentage of responses at level 1, both at 0.6% (6/972). The proportion of responses at level 2 (disagree) was the next lowest, ranging from 5.7% (55/972) for item 5 to 8.1% (79/972) for item 8. Some level 3 (undecided) and level 4 (agree) responses were close to each other compared with the distribution of the other levels. Level 4 had the highest percentage of responses and was the highest for item 5 (514/972, 52.9%). It is important to note that this pattern did not hold for item 8, as the proportion of responses at level 3 was higher than that at level 4 (414/972, 42.6%).

### Internal Consistency Reliability and Item Statistics

Table 2 shows that the reliability of the scale was ideal, as the Cronbach α was 0.92 and the split-half reliability was 0.88. In addition, the results of the Cronbach α if item deleted analysis indicated that all items should be retained and that the item-total correlation coefficient was between 0.715 and 0.781. Item 8 was rated as the most difficult item (mean score of 3.46), whereas item 5 was rated as the easiest (mean score of 3.68). There was no floor or ceiling effect on any of the items.

**Table 2:** Internal consistency reliability and item statistics of the Chinese version of the eHealth Literacy Scale.

	Internal consistency reliability^a^			Item statistics		
	α if item deleted	Item-total correlation	Score, mean (SD)		Floor (n=972), n (%)	Ceiling (n=972), n (%)
Item 1^b^	.915	0.715	3.58 (0.78)		9 (0.9)	87 (9)
Item 2^c^	.912	0.750	3.57 (0.80)		8 (0.8)	94 (9.7)
Item 3^d^	.910	0.772	3.63 (0.79)		8 (0.8)	105 (10.8)
Item 4^e^	.914	0.725	3.61 (0.81)		6 (0.6)	106 (10.9)
Item 5^f^	.910	0.781	3.68 (0.77)		7 (0.7)	106 (10.9)
Item 6^g^	.914	0.725	3.59 (0.77)		7 (0.7)	92 (9.5)
Item 7^h^	.914	0.723	3.57 (0.79)		6 (0.6)	100 (10.3)
Item 8^i^	.914	0.727	3.46 (0.79)		10 (1)	73 (7.5)
Overall	N/A^j^	N/A	28.69 (5.08)^k^		4 (0.4)	42 (4.3)

^a^Cronbach α=.92; split-half reliability=0.88.

^b^I know what health resources are available on the internet.

^c^I know where to find helpful health resources on the internet.

^d^I know how to find helpful health resources on the internet.

^e^I know how to use the internet to answer my questions about health.

^f^I know how to use the health information I find on the internet to help me.

^g^I have the skills I need to evaluate the health resources I find on the internet.

^h^I can tell high-quality health resources from low-quality health resources on the internet.

^i^I feel confident using information from the internet to make health decisions.

^j^N/A: not applicable.

^k^The overall scores of the Chinese version of eHealth Literacy Scale range between 8 and 40.

### Construct Validity and Convergent Validity

As presented in Tables 3 and 4, the results of the CFA showed that the 1-factor model had a poor fit, with high *χ*^2^/*df* (16.325) and RMSEA (0.126). However, the standardized factor loadings for the 1-factor model were high, ranging from 0.751 to 0.811, indicating good item properties. The standard factor loadings ranged from 0.769 to 0.834 for the 2-factor-1 model and from 0.761 to 0.825 for the 2-factor-2 model. Similar to the 1-factor model, both models suggested good fits based on the SRMR, CFI, and TLI, whereas RMSEA and *χ*^2^/*df* showed poor fits. For 3-factor-1 model, the standard factor loadings ranged from 0.786 to 0.844, and among all 5 models, only this model had all the fit indicators that met the suggested cutoff values (*χ*^2^/*df*=4.768, RMSEA=0.062, SRMR=0.020, CFI=0.987, TLI=0.979). Examination of the 3-factor-2 model revealed ideal SRMR, CFI, and TLI, but high *χ*^2^/*df* and RMSEA values.

The results of the analysis of convergent validity are reported in Multimedia Appendix 3. C-eHEALS 1-dimensional factor structure models, two 2-factor models, and two 3-factor models all had standardized factor loadings of more than 0.50, AVE values >0.50, and CR values >0.70, indicating good convergent validity.

**Table 3:** Confirmatory factor analysis of 5 solutions for Chinese version of the eHealth Literacy Scale (part 1).

1-factor^a^		2-factor						3-factor				
Item	Estimate	2-factor-1^b^		2-factor-2^c^			3-factor-1^d^				3-factor-2^e^	
		Subdomain	Estimate	Subdomain	Estimate	Subdomain			Estimate	Subdomain		Estimate
1	0.753	Knowledge about resources	0.769	Information seeking	0.761	Awareness			0.805	Information awareness		0.807
2	0.787	Knowledge about resources	0.803	Information seeking	0.795	Awareness			0.844	Information awareness		0.841
3	0.811	Knowledge about resources	0.834	Information seeking	0.822	Skills			0.834	Information seeking		0.817
4	0.766	Knowledge about resources	0.777	Information seeking	0.773	Skills			0.786	Information seeking		0.814
5	0.819	Knowledge about resources	0.817	Information seeking	0.819	Skills			0.827	Information engagement		0.752
6	0.753	Evaluation of resources	0.814	Information seeking	0.742	Evaluate			0.814	Information engagement		0.787
7	0.751	Evaluation of resources	0.821	Information appraisal	0.825	Evaluate			0.821	Information engagement		0.790
8	0.754	Evaluation of resources	0.807	Information appraisal	0.822	Evaluate			0.807	Information engagement		0.789

^a^In accordance with the model proposed by Norman and Skinner [14].

^b^In accordance with the model proposed by Richtering et al [30]. Corresponding to the item number in the 1-factor model, items 1 to 5, 6 to 8 belong to the knowledge about resources and the evaluation of resources subdomain, respectively.

^c^In accordance with the model proposed by Soellner et al [29]. Corresponding to the item number in the 1-factor model, items 1 to 5 and 8, 6, and 7 belong to the information seeking and information appraisal subdomains, respectively.

^d^In accordance with the model proposed by Sudbury-Riley et al [33]. Corresponding to the item number in the 1-factor model, items 1 and 2, 3 to 5, and 6 to 8 belong to the awareness, the skills, and the evaluate subdomains, respectively.

^e^In accordance with the model proposed by Paige et al [35]. Corresponding to the item number in the 1-factor model, items 1 and 2, 3 and 5, 4 and 6 to 8 belong to the information awareness, the information seeking, and the information engagement subdomains, respectively.

**Table 4:** Confirmatory factor analysis of 5 solutions for Chinese version of the eHealth Literacy Scale (part 2).

1-factor^a^			2-factor			3-factor	
		2-factor-1^b^		2-factor-2^c^	3-factor-1^d^		3-factor-2^e^
Chi-square (*df*)	326.499 (20)	119.551 (19)		241.950 (19)	81.051 (17)		210.129 (17)
Root mean square error of approximation	0.126	0.074		0.110	0.062		0.108
Standardized root mean squared residual	0.043	0.020		0.039	0.020		0.036
Comparative fit index	0.938	0.980		0.955	0.987		0.961
Tucker-Lewis index	0.913	0.970		0.934	0.979		0.936

^a^In accordance with the model proposed by Norman and Skinner [14].

^b^In accordance with the model proposed by Richtering et al [30]. Corresponding to the item number in the 1-factor model, items 1 to 5, 6 to 8 belong to the knowledge about resources and the evaluation of resources subdomain, respectively.

^c^In accordance with the model proposed by Soellner et al [29]. Corresponding to the item number in the 1-factor model, items 1 to 5 and 8, 6, and 7 belong to the information seeking and information appraisal subdomains, respectively.

^d^In accordance with the model proposed by Sudbury-Riley et al [33]. Corresponding to the item number in the 1-factor model, items 1 and 2, 3 to 5, and 6 to 8 belong to the awareness, the skills, and the evaluate subdomains, respectively.

^e^In accordance with the model proposed by Paige et al [35]. Corresponding to the item number in the 1-factor model, items 1 and 2, 3 and 5, 4 and 6 to 8 belong to the information awareness, the information seeking, and the information engagement subdomains, respectively.

### Content Validity

A panel of experienced experts, including a professor, an associate professor, a graduate student, and 2 doctoral candidates with research expertise in health care, assessed the relevance of the 8 items in the scale. Items were rated on a 4-point Likert scale, where 1 indicated “not relevant,” 2 indicated “marginally relevant,” 3 indicated “quite relevant,” and 4 indicated “strongly relevant.” The I-CVI was calculated from the experts’ relevance ratings for items 3 or 4, whereas the S-CVI/Ave was derived by averaging the proportional relevance judgments of all experts (Multimedia Appendix 4). Although S-CVI/Ave was slightly below 0.90, all 5 experts had I-CVIs equal to or above 0.80, indicating strong content validity for the 3-factor model [57].

### Measurement Invariance

Table 5 describes the entire validation process of the cross-gender invariance measurement test based on the 3-factor model of Sudbury-Riley et al [33].

**Table 5:** Fit statistic summary for testing measurement invariance in the 3-factor solution of Sudbury-Riley et al [33].

Model	Chi-square (*df*)	Normed fit index	Incremental fit index	Tucker-Lewis index	Comparative fit index	Relative fit index	Root mean square error of approximation	Akaike information criterion	Model comparison, Δ*χ*^2^ (Δ*df*)^a^
Model 1^a^	98.145 (34)	0.981	0.987	0.979	0.987	0.968	0.044	174.145	N/A^b^
Model 2^c^	101.124 (39)	0.980	0.988	0.982	0.988	0.971	0.041	167.124	2.979 (5)
Model 3^d^	113.937 (45)	0.978	0.986	0.983	0.986	0.972	0.040	167.937	15.792 (11)
Model 4^e^	176.239 (53)	0.965	0.975	0.974	0.975	0.963	0.049	214.239	78.094^f^ (19)

^a^Model 1 is the configural model.

^b^N/A: not applicable.

^c^Model 2 is the measurement model.

^d^Model 3 is the structural model.

^e^Model 4 is the error variance invariance model.

^f^*P*<.001.

First, as stated in the Statistical Analysis section, the baseline model was successful in establishing configural invariance. Second, the measurement model was built on a configural model that constricted the equality of factor loadings between the 2 gender groups. The change in *χ*^2^/*df* was not statistically significant, and the results (ΔNFI=−0.001, ΔIFI=0.001, ΔTLI=0.003, ΔCFI=0.001, ΔRFI=0.003, ΔRMSEA=−0.003) supported the factor loadings of each latent variable between different groups being comparable, thus implying that the metric in variance (or weak invariance) was established. Furthermore, based on the measurement model, the structural model hypothesized that the factor variances and covariances are equal across genders. The results showed that the model did not deteriorate significantly (ΔNFI=−0.003, ΔIFI=−0.001, ΔTLI=0.004, ΔCFI=−0.001, ΔRFI=0.004, ΔRMSEA=−0.004), again the change in *χ*^2^/*df* was not statistically significant, invariance was supported. Finally, we tested whether the error variance invariance had a cross-group equivalence. The change in chi-square (*χ*^2^/*df*=4.110) was statistically significant at *P*<.001, implying that the error variance invariance was rejected. Moreover, the Akaike information criterion value for the measurement model (value=167.124) was lower than that of the models testing for configural (value=174.145) invariance, and the Δ values were well below the cutoff values (ΔNFI=−0.016, ΔIFI=−0.012, ΔTLI=0.005, ΔCFI=−0.012, ΔRFI=0.005, ΔRMSEA=0.005). Taken together, these results partially support strict invariance.

## Discussion

### Principal Findings

C-eHEALS is a valid and reliable instrument, unlike the previous validation of the Lily and 2-factor models that have been validated in the Chinese population [23,38,39,50]. We found that it is possible to have a multifaceted structure in the Chinese population, and after comparing the 5 representative models, the 3-factor model yielded the best results. This model responds to the following 3 factors: awareness, skills, and evaluate [33]. As eHEALS has received much attention, it has faced validity controversies [63-66], and because of this, research evidence of invariance measurement across countries is necessary when making cross-national comparisons [42-48]. To our knowledge, this is the first invariance measurement study conducted on C-eHEALS and contributes research evidence to support the measurement invariance of gender. These findings will help organizations revert to original social cognitive theory base of Norman and Skinner [14] in their strategy development to ensure that eHealth resources are developed and used as effectively as possible.

### Comparison With Prior Work

In contrast to our undergraduate sample, the sample in a previous Chinese study was drawn from inpatients [38], chronic patients [50], general community residents [39], and rural populations [23]. In addition to the fact that our sample of undergraduates is different from the abovementioned studies, an important reason is that this sample is somewhat representative of the young adult population in China and has the characteristic of being active in web-based information searches.

In terms of study content, as we previously noted, the C-eHEALS multidimensional factor structure has not received much attention from Chinese researchers; therefore, we only found comparable results in the study by Xu et al [38]. First, the same situation appeared in both studies: in the fit analysis of the unidimensional structural model, *χ*^2^/*df* and RMSEA were unsatisfactory, whereas the values of SRMR were acceptable (whether the model was revised or not) [53-55]. Second, with the 2-factor solution of knowledge about resources versus evaluation of resources in eHEALS, the RMSEA of this study was acceptable but showed worse fit results in their initial 2-factor model [38]. Similar to the unidimensional model, *χ*^2^/*df* was unsatisfactory in both studies, whereas the SRMR values were acceptable. The study by Xu et al [38] showed that the revised 2-factor model showed a better fit, and as this study did not involve model modification, we roughly compared the superior model of this study (3-factor-1) with it and found that the fit indices of the 2 models were relatively close.

This study provides preliminary evidence of invariance measures for the 3-factor structure of C-eHEALS. However, this cannot be compared with the results of other Chinese studies. Other studies on measurement invariance in languages other than the English language also considered invariance measures across gender, education level, and age groups [42-44].

In addition, this study follows the factor structure proposed by Sudbury-Riley et al [33], and it is important to note that the last nested invariance measurement model is different in the 2 studies; specifically, in their work, scalar invariance tests means factor loadings and intercepts are constrained, whereas the third model in our procedure is the error variance invariance model. As previous studies have pointed out, full invariance is difficult to obtain [49]. Although the error variances associated with each observed variable item are also part of the measurement model, testing their equality across groups is considered too stringent and is therefore rarely implemented [59]. Even so, although the change in *χ*^2^ was significant in the last model tested in both studies, evidence for strict invariance is supported, considering that the changes in the other fit metrics were within the permissible range [61,62].

### Limitations

Using undergraduates as a representative group of young adults loses the significance of the contrast from differences in educational attainment, as well as the contrast between urban and rural areas among young adults in China. Convenience and snowballing sampling methods may lack rigorous control of random sampling techniques, and the lack of control over participant selection may compromise the study’s validity. The study’s reliance on self-reported data may have introduced response biases, and in this study, social desirability bias was not negligible. As too few participants chose paper-and-pencil surveys in our mixed-mode surveys, we were not confident in conducting an invariance measurement test of paper-and-pencil versus electronic surveys. However, based on the popularity of electronic surveys, such tests are necessary. Furthermore, various invariance measurement test procedures will need to be carefully considered in future studies to make them more comparable with each other. More importantly, the translated version of the eHEALS as a measurement instrument may encounter language barriers that potentially affect respondents’ comprehension and responses. Misinterpretations or misunderstandings because of linguistic differences may limit cross-cultural generalizability, thereby not fully capturing the cultural intricacies in the findings.

### Conclusions

The 3-factor structure of awareness, skills, and evaluate, based on a combination of social cognitive and self-efficacy theories, can inspire health care practitioners and researchers regarding the development of interventions for eHealth literacy. This study provides evidence of measurement invariance across genders under the factor structure described above, providing evidence that variation in scores are due to differences in the structure and not due to differences in other confounding variables.

## Appendix

Multimedia Appendix 1 The Chinese version of the eHealth Literacy Scale and the sociodemographic question items.

Multimedia Appendix 2 Data set.

Multimedia Appendix 3 Convergent validity analysis of 5 solutions for Chinese version of the eHealth Literacy Scale.

Multimedia Appendix 4 Content validation form.

## Abbreviations

AVE: average variance extracted
C-eHEALS: Chinese version of eHealth Literacy Scale
CFA: confirmatory factor analysis
CFI: comparative fit index
CR: composite reliability
eHEALS: eHealth literacy scale
I-CVI: item-level content validity index
IFI: incremental fit index
NFI: normed fit index
RFI: relative fit index
RMSEA: root mean square error of approximation
S-CVI/Ave: average of the item-level content validity index scores of all items on the scale
SRMR: standardized root mean squared residual
TLI: Tucker-Lewis index
