# Supplementary material for: Structural Validation and Measurement Invariance Testing of the Chinese Version of the eHealth Literacy Scale Among Undergraduates: Cross-Sectional Study
Source: J Med Internet Res. 2023 Dec 13;25:e48838. doi: 10.2196/48838 (PMC10753429; doi:10.2196/48838)
Supplement: Multimedia Appendix 4 [file jmir_v25i1e48838_app4.docx]

1) Content Validation Form

| **Content Validation Form** | |
| --- | --- |
| Dear Experts，  This study's objective was to evaluate the C-eHEALS, the Chinese version of the eHealth Literacy Scale, in terms of its measurement properties.  The list contains 3 dimensions and 8 items related to Chinese version of the eHealth literacy scale, and we look forward to your professional judgment on the relevance of each question to its domain of measurement. Your review should be based on the definitions and relevant terminology we provide. Please be as objective and constructive as possible in your review comments and rate them using the form we have provided below.  **The following criteria should be followed for scoring relevance：**  **1=This question is not relevant to the measurement dimension**  **2=The question is marginally relevant to the measurement dimension**  **3=The question is quite relevant to the measurement dimension**  **4=The question is strongly relevant to the measurement dimension** | |
| **Dimension 1:** *Awareness*  **Description:** *The first factor comprises items relating to knowledge about health resources and information that are available on the Internet.* | |
| 1.Grocery store or convenient store deals on sugary drinks, like buy-one-get-one free and other sales, are designed to get people addicted to sugar | 1. ② ③ ④ |
| 2. Sugary drink companies are very powerful, even outside of the beverage business | 1. ② ③ ④ |
| **Dimension 2:** *Skills*  **Description:** *The second factor relates to the skills needed to access and use the health resources and information.* | |
| 3. I know how to find helpful health resources on the Internet. | 1. ② ③ ④ |
| 4. I know how to use the Internet to answer my questions about health. | 1. ② ③ ④ |
| 5. I know how to use the health information I find on the Internet to help me. | 1. ② ③ ④ |
| **Dimension 3:** *Evaluate*  **Description:** *The third factor relates to levels of self-belief in the ability to use this information effectively.* | |
| 6. I have the skills I need to evaluate the health resources I find on the Internet. | 1. ② ③ ④ |
| 7. I can tell high-quality health resources from low-quality health resources on the Internet. | 1. ② ③ ④ |
| 8. I feel confident using information from the Internet to make health decisions. | 1. ② ③ ④ |

2) The relevance ratings on the item scale by five experts

| **The relevance ratings on the item scale by nine experts** | | | | | | | |
| --- | --- | --- | --- | --- | --- | --- | --- |
| Item | E1 | E2 | E3 | E4 | E5 | Experts in agreement | I-CVI |
| Q1 | 1 | 1 | 1 | 1 | 1 | 5 | 1 |
| Q2 | 1 | 1 | 1 | 0 | 1 | 4 | 0.8 |
| Q3 | ·0 | 1 | 1 | 1 | 1 | 4 | 0.8 |
| Q4 | 1 | 1 | 1 | 1 | 0 | 4 | 0.8 |
| Q5 | 0 | 1 | 1 | 1 | 1 | 3 | 0.8 |
| Q6 | 1 | 1 | 1 | 1 | 1 | 5 | 1 |
| Q7 | 1 | 1 | 1 | 1 | 1 | 5 | 1 |
| Q8 | 1 | 1 | 0 | 1 | 1 | 4 | 0.8 |
| Proportion  of relevance |  |  |  |  |  | S-CVI/Ave | 0.88 |
|  | 0.75 | 1 | 0.88 | 0.88 | 0.88 |  |  |
